# Executive/life coaching for first year medical students: a prospective study

**DOI:** 10.1186/s12909-019-1564-4

**Published:** 2019-05-22

**Authors:** Donna Cameron, Laura J. Dromerick, Jaeil Ahn, Alexander W. Dromerick

**Affiliations:** 1 Department of Family Medicine, Georgetown University, 4000 Reservoir Road NW, Washington, DC 20007 USA; 2 LJD Coaching, 1579 44th Street NW, Washington, DC 20007 USA; 3 Department of Biostatistics, Bioinformatics & Biomathematics, Georgetown University, 4000 Reservoir Road NW, Washington, DC 20007 USA; 4 Department of Rehabilitation Medicine, MedStar National Rehabilitation Network, Georgetown University, 102 Irving Street NW, Washington, DC 20010 USA

**Keywords:** Students, Medical, Curriculum, Coaching, Education, Medical, Mentoring, Evaluation studies, Resilience, Preclinical education, Life coaching, Executive coaching

## Abstract

**Background:**

Student physicians are particularly prone to high rates of poor mental and physical quality of life, including depression, anxiety, and fatigue. We prospectively tested whether a structured, theory-based executive/life coaching program tailored to first year medical students in the United States was feasible, tolerable, and would be recommended by participants. Secondary goals included impact on coaching goals, resilience, and perceived stress.

**Methods:**

This single-arm intervention study evaluated a program of two group and two private coaching sessions during the first year, second semester of the Georgetown University School of Medicine Class of 2019. Survey data (global and tailored questions, Connor-Davidson resilience scale, Friedricksson-Larsson stress question) were collected from participants at baseline and post-intervention.

**Results:**

37/40 students completed the intervention; 32 completed the pre-post surveys. Most (32/37) were willing to recommend the program (16/37 were very willing) and 29/37 recommended inclusion in the curriculum. Responses to tailored questions showed significant increases in self-efficacy regarding stress management (*p* < 0.001); increased awareness of thoughts about stress and management of those thoughts (*p* = 0.05). Reported improvements in time management (*p* = 0.10) and energy for relationships and school (*p* = 0.089) did not achieve significance. Global resilience rating was not different (*p* = 0.186), but significant changes were seen in control (*p* = 0.029) and spiritual influence (*p* = 0.005) factors. Although the Friedricksson-Larsson item was not significantly different (*p* = 0.242), 40.6% of participants reported decreased stress and 40.6% reported unchanged stress during this most challenging preclinical semester. Substantial ceiling effects were seen in study measures.

**Conclusions:**

We showed that a tailored executive/life coaching program for first year medical students in the United States is feasible, tolerable, and safe; adherence was excellent. Global utility ratings and willingness to recommend coaching provide substantial support for efficacy. Better measures and larger-scale clinical trial designs are needed for formal proof.

## Background

Student physicians can be susceptible to increased rates of poor mental and physical quality of life, including depression, anxiety, and fatigue [1]. These challenges are associated with higher dropout rates, suicidal ideation, and substance abuse. Compromises in professionalism have also been noted. Students can be less likely to hold altruistic views about a physician’s societal responsibilities, more likely to engage in unprofessional behaviors (misreporting test results, plagiarism) and less likely to correctly identify commercial conflicts of interest [2]. Thus, an effective, feasible, and affordable method is needed to manage the stressors associated with medical school and set behavioral and attitudinal patterns for the career that follows [3, 4].

Executive/life coaching may offer an approach. Coaching is an intervention that is intended to focus on “performance, learning, and development [5]” but is not focused on acquiring specific technical skills or knowledge, such as teaching the performance of a surgical procedure [6]. Trained and certified professional coaches apply non-directive techniques that help the individual to articulate their own concerns and devise their own solutions. Coaches use positive psychology, active listening, creating awareness, designing actions, planning, goal setting, and managing progress and accountability [7] to bring the individual to a state of awareness and action. Moreover, a professional coach can be an outsider, eliminating concerns about privacy, professional evaluation, and professional stigma, and allowing more authentic communication about problems, challenges, and fears. Coaching is directed at personal and professional growth and is therefore appropriate for individuals experiencing transitions, including new medical students [8, 9].

Coaching is applied in many settings, particularly in the corporate, business, and executive settings. A recent review of the academic literature on coaching shows that it began in the 1930’s, focusing first on manufacturing management. There has been a logarithmic growth in literature during the last few years, and coaching is now widely applied to improve the performance of individuals in many fields [10]. The first randomized, controlled trial of executive coaching showed that public health executives who were coached by professional coaches significantly increased goal attainment, resilience, professional well-being, and reduced stress and depression [11]. In the study’s qualitative evaluation, participants reported “coaching helped increase self-confidence and personal insight, build management skills and helped participants deal with organisational change.”

A recent meta-analysis on coaching revealed that it is effective within large organizations in five domains: performance/skills, well-being, coping, work attitudes, and goal-directed self-regulation [7]. Effect sizes were substantial, ranging from small but educationally significant (*g* = 0.43) for coping, to quite large (*g* = 0.74) for goal-directed self-regulation. Investigators in Canada, New Zealand, Great Britain and Australia have been at the forefront in applying the principles and techniques of coaching to physicians and medical students [8, 12, 13]. Canadian family practitioners identified key areas they attributed to physician success and resilience [13]: attitudes and perspectives (including self-awareness, and accepting personal limitations); balance and prioritization (including setting limits, effective educational approaches, and honoring the self); and supportive relations (including positive personal relationships, effective professional relationships, and communication skills [13]). More recently [3], other groups in the US have begun to focus on related issues [14–16].

Our goal was to explore whether a theory-based, structured executive life coaching tailored to the needs of first year (M1) medical students could provide useful tools and approaches to manage the high demands and psychosocial stress of medical training. Our rationale was that with this additional support, M1 students could be more successful in preclinical learning and presumably evolve to become more effective and collaborative in a clinical career expected to become increasingly complex and demanding [17]. In this pilot study, we hypothesized that students who participated in the program would demonstrate tolerability by completing the program, report utility of the intervention through a willingness to recommend the intervention, and report increased stress management skills and social resilience.

## Methods

### Subjects

This prospectively designed, single arm study was approved by the Georgetown University Institutional Review Board, and all participants provided written informed consent. Participants were volunteers from second semester, first year medical students (M1) from the Class of 2019 at the Georgetown University School of Medicine. In that class of 196, 53% were female and the average age was 24 (range 21–38).

### Procedures

**Fig. 1:**
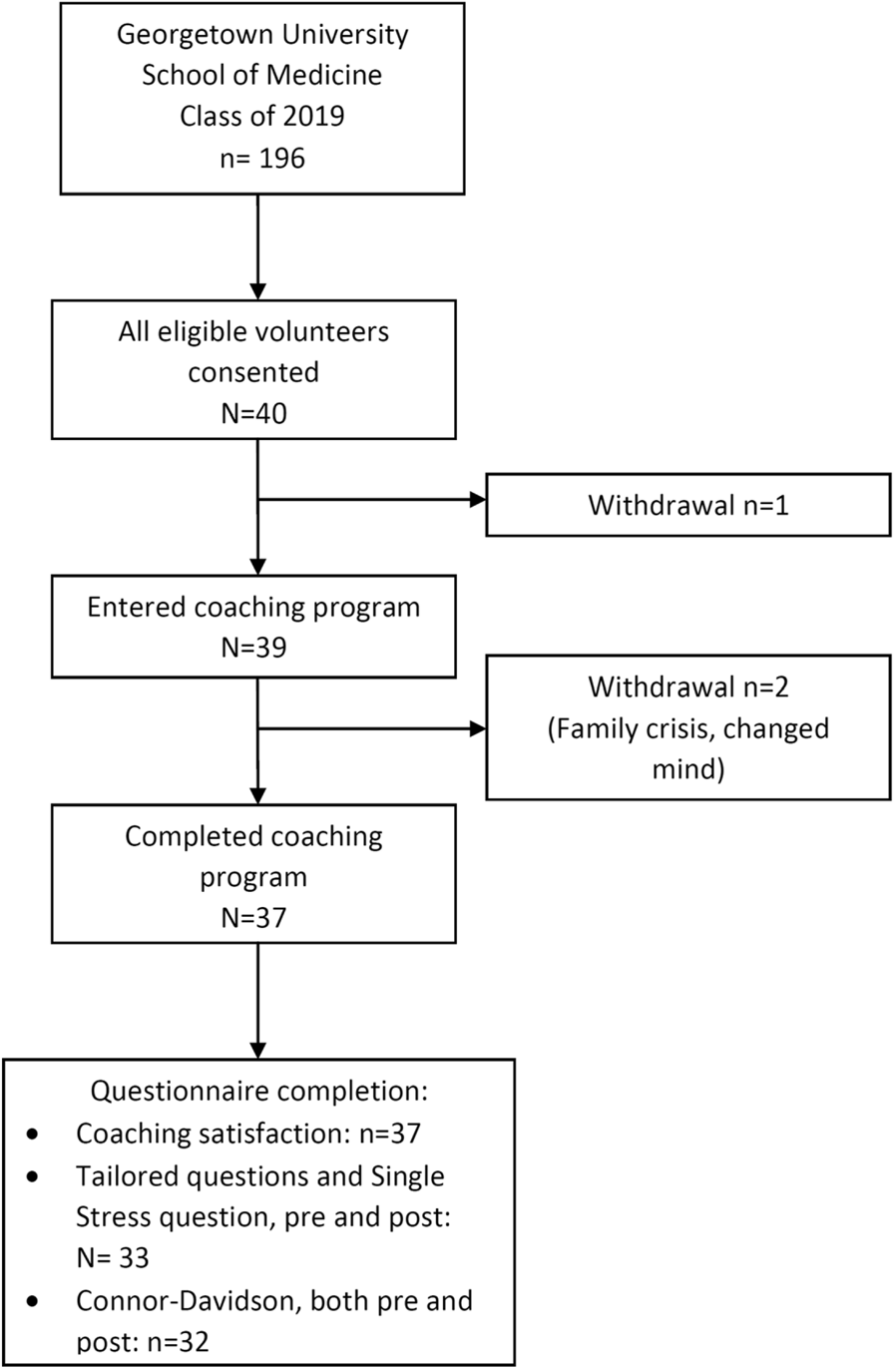
CONSORT Diagram

We chose the second semester of the first year of medical school for this intervention because the initial stressors associated with transitioning to medical school (moving to a new city, new roommates, etc.) had passed. Moreover, in the Georgetown curriculum the second- semester Neuroscience and Head & Neck courses are deemed particularly challenging. Self-selected volunteers from the Class of 2019 were elicited through announcements, informational sessions, flyers, and email. Forty-one students volunteered; 1 was ineligible and consent was obtained from 40. See Fig. 1, for the study CONSORT diagram.

The coaching intervention emerged from an M1 selective course addressing stress management and school-life balance [18] (led by DC). The coaching was provided by two professional executive coaches, one of whom (LJD) was clinically trained and had > 25 years experience coaching physicians, resident physicians, and academics [19]. A clinical trialist experienced in behavioral intervention trials (AWD) assisted with standardizing and documenting the intervention and with study design; the study biostatistician (JA) was consulted to develop and execute a prespecified analysis plan.

The intervention consisted of two group sessions alternating with two individual sessions, totaling four interactions over the semester. In the group sessions, concepts were introduced, including life vision and goals, and managing stress. Table 1 displays the content of the group and individual sessions. Participants were asked to use a variety of coaching tools to clarify life goals, develop a life vision and goals, reframe negative thoughts, and create strategies for decision-making. In the one hour one-on-one sessions, issues specific to the individual were discussed with the coach. To maximize the quality and authenticity of interactions, the same coach met with the same individual participant whenever possible, and the participants knew that no coaching content would be shared with faculty or administration. For two participants, a group session was missed (one due to illness, one to participate at a research conference) but interactions equivalent to the missed group sessions were provided.

**Table 1:** Overview of M1 Coaching Program

Session and Type	Goals
1. Vision and Goals, Group Format (10 per group, 4 groups, 90 min/group)	• Articulate and clarify long term career vision
	• Create short- term goals to overcome academic challenges
	• Access goals when needed
	• Maintain prominence of goals in daily activities and long term planning
	• Apply goals to daily planning
2. Visioning and goals, 60 min individual session	• Build relationship with student
	• Identify personal strengths and values
	• Break goals down into attainable steps
	• Create action plan for goals
	• Identify and anticipate individual barriers, strategies to overcome
3. Managing stressors, Group format (10 per group, 4 groups, 60 min/group)	• Stressors common to all participants
	• Reframing negative thinking
	• Chunking and scheduling to improve time management
	• How to make decisions
	• Supporting personal confidence
	• Rebounding from failures, real or perceived
4. Managing stressors, 60 min individual session	• Conserving energy
	• Identifying and approaching individual’s negative thinking
	• Individual application of decision-making and prioritization tools
	• Application of techniques throughout medical school career

### Measures

Our primary study outcomes were the feasibility, tolerability, and safety of the intervention. These were evaluated by protocol adherence (number of sessions attended), number of adverse events, and willingness to recommend the intervention to others and for inclusion into the M1 curriculum. In the tailored survey tool, we included several opportunities to prompt free text feedback about the program.

Since we could not identify well-developed, coaching-specific evaluation scales relevant to medical education, we developed a number of questions tailored to the circumstances of M1 students and to the goals of the coaching intervention. These questions used 4-level Likert ratings and addressed the following issues: managing stress, creating a future vision, time management, self-awareness, and energy. These questions are listed in Table 2. After the intervention we also surveyed participants’ assessment of the value and helpfulness of the group and individual sessions, as well as their willingness to recommend the intervention.

**Table 2:** Tailored interview questions

Managing Stress	
How confident do you feel in your ability to handle stress?	
How confident are you that you have the tools to manage stress?	
Creating a future vision	
Given your current medical school experience, how helpful is it to imagine your life in ten years?	
Given your current medical school experience, how helpful is it to set goals for your future?	
Given your current medical school experience, how helpful is it to create a plan for your future?	
Goal Setting	
How valuable is it to identify specific career goals (e.g., residency)?	
How valuable is it to identify specific personal goals (e.g. lifestyle)?	
Time Management	
How well are you able to set priorities?	
How well do you manage your time?	
Self-awareness of stress management strategies	
How adept are you at noticing the way you think about stress?	
How adept are you at managing your thoughts about stress?	
Energy	
How high is your energy for relationships with friends and loved ones?	
How high is your energy for medical school demands?	
How high is your optimism about school?	

Participants rated responses from 1 (very confident/helpful/valuable/well/adept/high) to 4 (not at all confident/helpful/valuable/well/adept/high)

To assess social resilience, we used the Connor-Davidson Resilient Beliefs Scale [20], a self-report scale rating personal qualities and beliefs “that enable one to thrive in the face of adversity.” The Scale’s high reliability, measured in a sample of the general population using Cronbach’s alpha, had an internal consistency of 0.95 and 0.93 in pre- and post- surveys, respectively. Test-retest reliability, measured by Guttman’s Lambda 6, is equally 1 in pre- and post surveys.

Using the Connor-Davidson Resilient Beliefs Scale, participants rated 25 items on a scale of one (not true at all) to 4 (true nearly all the time) for a total score of up to 100; higher scores indicate greater resilience. Five scale factors have been identified [18]: (1) Personal competence/tenacity/high standards; (2) Trust in one’s instinct/tolerance of negative affect; (3) Positive acceptance of change/secure relationships; (4) Control, and (5) Spiritual influences. The 25th question (“pride in your achievements”) was inadvertently deleted in the study materials, so analyses include 24 items.

To assess overall perceived stress, we used the Friedricksson-Larsson single question evaluation [21]: “Stress means a situation when a person feels tense, restless, nervous, or anxious, or is unable to sleep at night because his or her mind is troubled all the time. Do you feel that kind of stress these days?” Participants responded on a 5-point Likert scale that ranged from 1 “not at all” to 5 “very much.”

### Analysis

All 37 individuals who completed the coaching intervention completed the survey about satisfaction regarding coaching, willingness to recommend, and inclusion in curriculum. Thirty-three of these also completed the tailored question survey, the Connor-Davidson scale, and the Single Stress Question at both time points, and were thus included in the per protocol survey data analysis. All analyses were conducted using Statistical Analysis System (SAS) software suite (ver 3.3).

Descriptive statistics of frequencies and 95% exact confidence intervals were summarized for survey data. For comparison analyses between pre- and post-intervention, we used 32 per-protocol pairs who completed both pre- and post- surveys in order to remove subject-specific correlations and potential confounders such as gender and age. For each of 39 ordinal items, we used the non-parametric Mann-Whitney U test and Cohen’s agreement kappa to examine the impact of the intervention on participants’ attitudes during the semester. To retain all information in ordinal outcome scales, we also fit an ordinal logistic regression model for shift analyses. We illustrated changes in frequencies of scales per each item using bar-plots with test statistics. We also performed paired t-tests to detect changes in the aggregated scores obtained from six subgroups of tailored interview questions (see Table 2); 1) managing stress, 2) future creation, 3) goal setting, 4) time management, 5) self-awareness, 6) energy of the six factors as in Table 1. We performed similar analyses for the five factors of the Connor Davidson survey: 1) competence, 2) trust in one’s instincts, 3) positive acceptance of change, 4) control, and 5) spiritual influence. Table 3 shows Pre-post changes in tailored survey questions. Two-sided *p*-value less than 0.05 were considered to be statistically significant.

**Table 3:** Pre-post changes in tailored survey questions (*n* = 33)

Category	Paired t-test	*P*-values
Managing Stress	3.917	< 0.001
Future creation	−0.235	0.815
Goal setting	0.414	0.681
Time management	1.677	0.103
Self-Awareness	2.030	0.05
Energy	1.75	0.089
Stress	1.190	0.242

All scores reported using a 4-point Likert scale, except question 40, which used a 5-point Likert scale. All scales ranged from 1 “not at all” to 4 (or 5) “very much”

## Results

Forty volunteers meeting criteria were consented and enrolled. Except for a modest over-representation of women, these were generally representative of the entire class: 72.5% female, mean age of 25.2 ± 3.0 years, and 33/40 (83%) entered their M1 year directly from undergraduate training.

Our primary endpoints of safety, feasibility and tolerability were well demonstrated. There were no protocol-related adverse events, and all participants completed the semester academically. Protocol adherence was excellent; 37/39 (94.9%) participants completed the full coaching program (2 group plus 2 individual interactions). (One participant was referred for professional counseling for anxiety and depression.) All 37 participants who completed the intervention found it to be of some value: 35/37 (94.6%) found it to be of average value or greater and only 2/37 (5.4%) found the program to be of limited value. Willingness to recommend the program to fellow students was high: 33 of the participants were willing to recommend this program to fellow students and 16 were very willing to recommend. Twenty nine participants recommended adding this program to the M1 curriculum. Additional topics requested were grouped into categories: career vision and strategy (10 requests), personal wellness (6 requests), career and communication skill development (3 requests), relationships and social life (2 requests), and finances (2 requests).

Regarding the format of the coaching intervention, both the group and the individual sessions were deemed valuable by most participants. Thirty participants reported group coaching to be helpful, both for its efficiency and because it showed them that their peers had similar challenges. Thirty four reported that the individual coaching format was helpful. The reported advantages of individual sessions were talking through challenges and receiving feedback (25 respondents), individualized goals and plans of action (15), and coaching by a coach who was not medical school faculty (3).

We analyzed the data from the tailored survey of the 32 participants who completed the intervention and both the pre- and post-intervention surveys. In the responses to the questions tailored to the goals of the intervention, we found highly significant effects in multiple domains targeted by the intervention, see Table 3. Most striking was the change in the participants’ self-efficacy regarding their ability to manage stress (*P* < 0.001); clearly the participants had gained confidence in this area. They also noted improvement in skills regarding stress management, reporting that they were now aware of their stress-related thoughts and their abilities and strategies to manage them (*P* < 0.05). Scores for time management skills increased, but these did not reach statistical significance (*P* = 0.10). Similar results were seen in reporting increased energy for personal relationships and school (*P* = 0.089). In marked contrast to the free text comments reported above, the survey questions did not detect benefit in coaching interventions focusing on the future (creating a vision for a future life, goal setting for the next year). This finding may be an artifact of the high initial scores in these domains.

The utility of the Connor-Davidson resilience scale was limited by a ceiling effect in measurement. Inspection of the scores on the individual items showed that baseline ratings on most items in the scale were already at or near the ceiling (86.5% of responses scored the top two categories at baseline). This indicates great stated confidence at baseline on the part of the participants, but also minimized detectable change in these domains. We saw no significant change in the scale’s summary score or in three of the individual factors that constitute the scale (see Table 4). However, highly significant changes were seen for the factors of Control and Spiritual influence (*P* = 0.005), suggesting that students became calmer and more accepting of events, and perceived more control over those events.

**Table 4:** Pre-post changes, Connor-Davidson Resilience Scale (*n* = 32)

Category	Paired t-test	*P*-values
Overall resilience, scale summary score	1.351	0.186
Factor 1: Personal competence, high standards, and tenacity.	−0.295	0.768
Factor 2: Trust in one’s instincts, tolerance of negative affect, and strengthening effects of stress	1.583	0.115
Factor 3: Positive acceptance of change, secure relationships	1.465	0.144
Factor 4: Control	2.220	0.029
Factor 5: Spiritual influence	2.893	0.005

We used the Friedricksson-Larsson single question evaluation of stress as a global evaluation of perceived stress; formal hypothesis testing showed no significant difference in the pre-post evaluation. However, lack of significance may be due to small sample size and lack of a comparison group: inspection of individual scores revealed that 13/32 respondents (40.6%) reported an improved stress level and 13/32 respondents reported no increase in stress despite this highly challenging period in the Georgetown curriculum.

## Discussion

The success of gifted and motivated students can be constrained by factors outside the classroom and laboratory; these are often poorly addressed or even acknowledged in medical school. These constraints include limited insight into career and life goals, inability to balance work and other priorities, nurturing personal relationships that support emotional resilience, and inability to structure time to accomplish specific tasks such as acquisition of particular clinical skills or of a body of knowledge (Delorio 2016). The coaching intervention in this study included teaching new skills for developing and evolving a vision of career and life goals, reframing of negative thoughts, and time management and prioritization. The impact of these skills may extend well beyond the M1 year into lifelong professional and personal satisfaction.

The 95% adherence rate in study participants provides strong and objective evidence of the high value that these stressed and busy individuals found in coaching. Further, their willingness to recommend the program to their classmates and for incorporation into the curriculum suggests that these participants valued the intervention so much that they were willing to publicly admit their own vulnerability in an attempt to help their peers, who are simultaneously their competitors. Both the group and individual coaching formats were praised. In free form text comments, the respondents were quite positive, and many requested an even more robust intervention. The questionnaire-based data have limitations, but generally also support the effectiveness of the coaching intervention.

We note that our study participants were self-selected and may not be representative of all Georgetown M1 students, and that Georgetown students may not be representative of a national sample. Our self-selected sample could have led to a group particularly primed to respond to coaching intervention, thereby overestimating the effect of coaching. The group choosing not to participate might have found coaching to be of no help, or even aversive. Alternatively, because this was not a randomized study, it could also be argued that the group that chose participation might be particularly self-aware, and that those who did not have enough insight to participate (or who were under too much self-perceived stress) might actually benefit even more from this intervention. Without an observation-only control group, we cannot separate coaching effects from non-specific effects of the M1 second semester experience. The safety of the coaching intervention could be even better confirmed by a longer follow-up period. The generalizability of our work to other coaching programs is unknown, nor can our study provide insight into which aspects of the intervention were efficacious.

We encountered three methodological challenges in measuring the effect of this executive/life coaching intervention. First, we were unable to locate published measures that we judged were both relevant and methodologically sound. In response, we selected a few well-known measures and also prospectively developed our own (albeit unvalidated) questionnaire tailored to the content of coaching. In this tailored questionnaire, several statistically significant changes were detected pre- and post-coaching, and the remaining questions were limited by ceiling effects. While most of the tailored survey findings are highly supportive of our hypotheses, confirmation of these findings will require further psychometric development of this or a similar tool.

The second challenge was that the medical student participants were quite different from the general population in their self-report of resilience. Our participants rated themselves so highly on the baseline Connor-Davidson scale that detection of improvement was almost impossible.

Third, our study’s pilot nature precluded key features of a pivotal clinical trial, including a large sample size and a control group. We interpret the finding that > 80% of participants reported improved or stable stress on the Friedricksson-Larson stress question as being supportive of coaching effectiveness. We speculate that the lack of worsening during this most challenging preclinical semester is evidence of effectiveness of our intervention. However, statistical significance was not achieved. Without a control group, we can only speculate on what the group’s outcome would have been without coaching.

Formal proof of efficacy will require a full-scale trial, and this study provides needed preliminary data. We note that the coaches in this study were trained and experienced professional coaches who were independent of the medical school faculty. Our study does not examine whether coaching using faculty (who are rarely formally trained in executive coaching and are responsible for grading performance and providing letters of recommendation) would be successful.

## Conclusion

In this prospectively designed pilot study, we evaluated the feasibility, safety, tolerability, and preliminary efficacy of an executive/life coaching intervention during the most challenging preclinical semester at the Georgetown University School of Medicine. We found that adherence was excellent; there were no safety issues. Nearly all study measures were improved or stable, despite the increased academic challenge of that semester. Further work will require measures that do not have pronounced ceiling effects and a study scope allowing a randomized controlled study design. Coaching may play an important role in the success of student physicians.

## Abbreviations

AD: Alexander Dromerick
DC: Donna Cameron
JA: Jaeil Ahn
LJD: Laura J. Dromerick
MS 1: First year medical student
SAS: Statistical Analysis Software
